# Association between health service use and diarrhoea management approach among caregivers of under-five children in Nepal

**DOI:** 10.1371/journal.pone.0191988

**Published:** 2018-03-01

**Authors:** Pramesh Raj Ghimire, Kingsley Emwinyore Agho, Andre M. N. Renzaho, Michael Dibley, Camille Raynes-Greenow

**Affiliations:** 1 School of Science and Health, Western Sydney University, Penrith, New South Wales, Australia; 2 School of Social Sciences and Psychology, Western Sydney University, Penrith, New South Wales, Australia; 3 Sydney School of Public Health, University of Sydney, Sydney, New South Wales, Australia; TNO, NETHERLANDS

## Abstract

**Introduction:**

Diarrhoea among children under-five is a serious public health problem in many developing countries, including Nepal. This study aimed to examine the association between health service utilization and diarrhoea management approaches among children under-five years in Nepal.

**Methods:**

The combined 2001, 2006 and 2011 Nepal Demographic and Health Survey (NDHS) data sets were examined and the sample included 2,655 children aged 0–59 months who had diarrhoea 2-weeks prior to the each survey. Multilevel logistic regression analyses that adjust for clustering and sampling weight were used to examine the association between health service utilization and diarrhoea management approaches (Oral Rehydration Solution, increased fluids and/or continued feeding).

**Results:**

The prevalence of extra fluids decreased significantly from 27% in 2001 to 15% in 2011 while that of ORS increased significantly from 32% in 2001 to 40% in 2011. The prevalence of continued feeding fluctuated between 83–89%. Multivariate analysis revealed that caregivers whose children received treatment or advice from health care providers during diarrhoea were 5.78 times more likely to treat diarrhoea with Oral Rehydration Solution (ORS) [adjusted Odds Ratio (aOR) 5.78, 95% confidence interval (CI) 4.50, 7.44], 1.56 (aOR 1.56, 95% CI 1.19, 2.05) times more likely to offer extra fluids, and 2.25 (aOR 2.25, 95% CI 1.50, 3.39) times more likely to use continued feeding than those who did not seek advice.

**Conclusions:**

Our findings indicate that health service utilization significantly improves diarrhoea management among under-five children. However, a broader national diarrhoeal disease control program to further reduce diarrhoea related morbidity and mortality in Nepal should focus on educating caregivers about the importance of the use of ORS as well as increase fluid intake to children under-five years with diarrhoea.

## Introduction

Globally, diarrhoea remains a leading cause of under-five mortality and morbidity, particularly in low-and middle-income countries including Nepal [1, 2, 3]. The 2015 global burden of disease study estimated that nearly half a million under-five deaths were caused by diarrhoea, and south Asia (including Nepal) stands second to sub-Saharan Africa with the highest number of these under-five deaths [3].

During the past three decades, international organizations such as World Health Organization (WHO) and the United Nations Children’s Fund (UNICEF) have proposed various management approaches for diarrhoea[4]. The first line approaches include: the use of oral rehydration solutions (ORS), increasing fluid intake, use of zinc supplements and continued feeding (including breastfeeding)[4, 5]. The impact of diarrhoeal disease control programs on childhood mortality have been documented in previous studies conducted in Egypt and the Philippines[6, 7]. These studies revealed that the decline in child mortality associated with diarrhoea may be due to increased use of ORS, extra fluids and continued feeding [6, 7], and other research concluded that diarrhoea management approaches are cost effective in reducing the overall burden of diarrhoea [8–14].

In Nepal, the national diarrhoeal disease control program emphasizes the use of the four treatment approaches for childhood diarrhoea; ORS, zinc supplementation, counselling on continued feeding to the caregivers, and the use of extra fluids; which are provided at all levels of the Nepalese health care system [15, 16]. Despite these initiatives, diarrhoea remains a public health concern, particularly in remote regions[17, 18]. Recently, the prevalence estimates for diarrhoea increased from 12% in 2006[19] to 14% in 2011[15]. Almost 50% of Nepalese children who experience diarrhoea do not have access to basic diarrhoea treatment approaches such as ORS or extra fluids[15].

There is substantial variation in uses around ORS, extra fluids and continued feeding as reported in the Nepal Demographic and Health Surveys of 2001, 2006 and 2011[15, 19, 20]. Hence, studies that examine the impact of health service use on ORS, continued feeding and/or extra fluids during childhood diarrhoea would provide important locally-relevant evidence to inform context-specific interventions geared towards reducing diarrhoea-related morbidity and mortality among children under-five years. Therefore, the aim of this study was to examine the association between health service use and diarrhoea management approaches among children aged 0–5 years in Nepal using nationally representative data from the Nepal Demographic and Health Survey (NDHS) for the years 2001, 2006 and 2011. This paper also provide insights into the three main diarrhoeal management approaches used by Nepalese government in order to be able to recommend the changes necessary for the successful implementation of the national diarrheal disease control program.

## Methods

### Data sources

The present study used nationally representative data from the Nepal Demographic and Health Survey (NDHS) for the period (2001–2011). The present analyses is based on publicly available NDHS datasets collected for the years 2001, 2006 and 2011[21]. Using multi-stage cluster sampling design, all NDHS collected data on various socio-demographic and health indicators including diarrhoea prevalence and its management approaches. The average response of three recent NDHS was 98.2% and the sample represents more than 98% of Nepal’s population. The details of survey methodology, sampling techniques and standard questionnaires are described elsewhere[15, 19, 20].

From 17,714 children aged 0–5 years (N = 6978 in 2001 NDHS[15], N = 5545 in 2006 NDHS[19], and N = 5391 in 2011 NDHS[20]), a sample of 2655 children (n = 1320 in 2001 NDHS, n = 624 in 2006 NDHS, and n = 711 in 2011 NDHS) who had diarrhoea 2 weeks prior the interviews of each survey were identified. The sample population was weighted to adjust for the multi stage cluster sampling effect.

### Outcome variables

In the NDHS, if a child had diarrhoea two weeks prior to each survey, mothers were asked how much a child was given to drink (including breastmilk), how much a child was given to eat, and was a child given a fluid made from ORS packets during the diarrhoea. The outcome variables are: (a) use of ORS, (b) use of increased fluids, (c) use of continued feeding (d) combination of all treatment approaches (ORS & extra fluids & continued feeding) and (e) combination of any treatment approaches (ORS or extra fluids or continued feeding) during recent diarrhoeal episodes. If a child had diarrhoea and was given fluid made from ORS packets, it was coded as 1, otherwise 0. If a child had diarrhoea and was given more liquids to drink, it was coded as 1, otherwise 0. If a child had diarrhoea and was given more, same as usual, or somewhat less food, it was coded as 1, otherwise 0.

### Exposure variable

The exposure variable of the study was derived from the women’s questionnaire for the section of immunization and health (Did you seek advice or treatment for the diarrhoea from any source?). The exposure variable was coded as 1 if the parents or carer of a child with diarrhoea sought treatment or advice from health care providers (except from pharmacies, shops and traditional practitioners), otherwise coded as 0.

### Potential confounding factors

**Fig 1:**
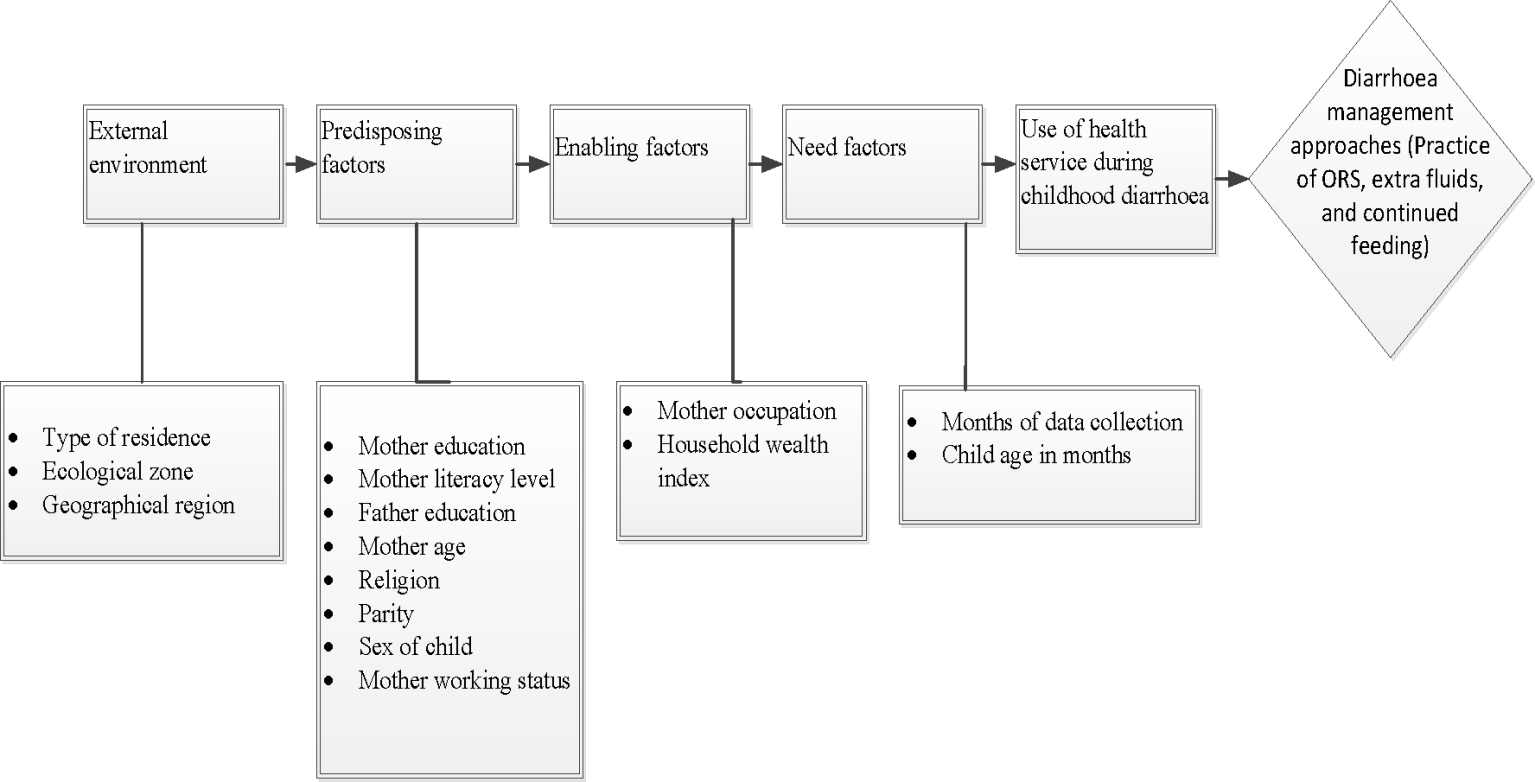
Conceptual framework for health service utilization and diarrhoea management approaches among children aged 0–5 years in Nepal, adopted from Anderson behavioural model.

The confounding factors examined in the study were based on the modified Anderson behavioural model[22] to examine the relationship between health service use and diarrhoea management approaches (Fig 1). We analysed 15 key confounding factors and they were classified as: external environment, predisposing factor, enabling factor and need factor. The external environmental factors consisted of: type of residence (Rural and Urban), ecological zone (Mountain, Hill and Terai), and geographical region (Eastern, Central, Western, Mid-Western and Far-Western). Nepal was divided into five Development Regions: Mid-Western, Western, Eastern, Central and Far-Western [15,19,20,23]. The Mid-Western Development Region comprised of three zones (Karnali, Bheri, Rapti) whereas, Western Development Region comprised of three zones (Gandaki. Lumbini, Daulagiri). Similarly, Eastern Development Region and Central Development Region covered three zones (Mechi, Koshi, Sagarmatha) and (Janakpur, Bagmati, Narayani), respectively. Far-Western Development Region only comprised two zones (Seti, Mahakali). Mid-Western, Western, Eastern, Central and Far-Western Development Regions covered 28%, 20%, 19%, 19% and 14%, respectively of the total land of Nepal [15,19,20,23]. The predisposing factors included mother’s current age, mother’s education, mother’s literacy level, father’s education, parity, mother’s religion, and mother’s working status. The enabling factors examined were mother’s occupation, household wealth index and the sex of the child. The household wealth index measures the economic status of the household. We used the wealth index factor scores as calculated by original DHS[15, 19, 20]. The combined original household wealth index factor scores were categorised into three: the bottom, 40% of households was referred to as poor households, the next 40% as the middle households and the top 20% as rich households, consistent with previous studies[24, 25].

### Statistical analysis

As part of the analysis, weighted frequency tabulation and percentage of study variables were first performed for exposure and all confounding factors. This was followed by univariate analyses that independently examined the association of all potential confounding and exposure variables. Multivariate analyses were used to examine the association between health service use and diarrhoea management approaches. As part of the multivariate analyses, staged modelling technique[26] was employed. As a process of staged hierarchical modelling technique, all external environmental factors were first entered into the baseline multivariable model with backward elimination to remove statistically non-significant variables (Model 1). Similarly, in the next stage, predisposing factors were examined with model 1 (Model 2). Next, enabling factors were assessed with model 2 (Model 3). Afterward, need factors were examined with model 3 (Model 4). In the final model (model 5), we examined the use of health service variable with the statistically significant environmental, predisposing, enabling, and need factors identified in the previous model. Variables significantly associated at the 5% significance level with each outcome measure were included in model 5 and reported in the study. We also tested collinearity and reported these findings. The analyses were performed using STATA (version 14.1). The Survey (SVY) function was applied, which allowed for adjustments for sampling weights for cluster sampling. We reported adjusted and unadjusted odds ratios and 95% confidence intervals.

### Ethics

The consent statement was read to each respondent in all three surveys and informed verbal consent from each respondent was signed by the interviewer. The Nepal Health Research Council (NHRC) in Kathmandu, Nepal and the ICF Institutional Review Board in Maryland, USA, approved all surveys. The first author sought and obtained permission from Measure DHS/ ICF International to use data as part of his doctoral dissertation within the School of Science and Health at Western Sydney University, Australia.

## Results

Of the 2655 children with diarrhoea, only 27% of their caregivers sought treatment or advice from health care providers (Table 1). Of the 27% who sought treatment or advice from the health care providers during diarrhoea, 17% used ORS and sought treatment and 10% sought treatment but did not use ORS. About half (46.5%) of the diarrhoea cases in the study occurred during the high diarrhoea prevalence period (April–August). The majority of children (92%) were rural residents, and 74% were at least two years of age. Nearly half (49%) of the children were from poor socioeconomic households.

**Table 1:** Characteristics of children under-five years of age with diarrhoea in Nepal, NDHS 2001–2011.

Study variables	n(%)	Study variables	n(%)
**Type of Residence**		**Parity**	
Rural	2443(92.0)	6+	332(12.5)
Urban	211(8.0)	(4–5)	505(19.0)
**Ecological zone**		(2–3)	1144(43.1)
Mountain	219(8.3)	1	674(25.4)
Hill	1030(38.8)	**Sex of child**	
Terai	1405(53.0)	Female	1220(46.0)
**Geographical region**		Male	1435(54.1)
Central	948(35.7)	**Mother working status (n = 2628)**	
Eastern	632(23.8)	Currently working	1829(68.9)
Western	480(18.1)	Currently not working	798(31.1)
Mid-western	312(11.8)	**Mother occupation (n = 2611)**	
Far-western	283(10.7)	Agriculture	1827(68.8)
**Mother education**		Non- agriculture	191(7.2)
No education	1723(64.9)	Not working	593(23.3)
Primary	442(16.7)	**Household wealth index**	
Some secondary to higher	489(18.4)	Poor	1300(49.0)
**Mother literacy level (n = 2651)**		Middle	577(21.7)
Cannot read at all	1567(59.0)	Rich	777(29.3)
Able to read	1084(40.8)	**Months of data collection**	
**Father education**		January- March	1420(53.5)
No education	1288(48.5)	April- August	1235(46.5)
Primary	869(32.7)	**Child age in months**	
Some secondary to higher	498(18.7)	(0–11)	690(26.0)
**Mother age**		(12–23)	819(30.9)
30–49	787(39.6)	(24–59)	1145(43.1)
20–29	1662(62.6)	**Use of health service during diarrhoea**	
<20	206(7.8)	No	1937(73.0)
**Religion**		Yes	718(27.0)
Buddhist	218(8.2)		
Hindu	2166(81.6)		
Others	271(10.2)		

n: Weighted counts. Other religion Includes mainly Christian, Muslims and Kirat; Non-agriculture occupation includes skilled and professional jobs; Counts and percentages vary between categories because of missing values.

**Fig 2:**
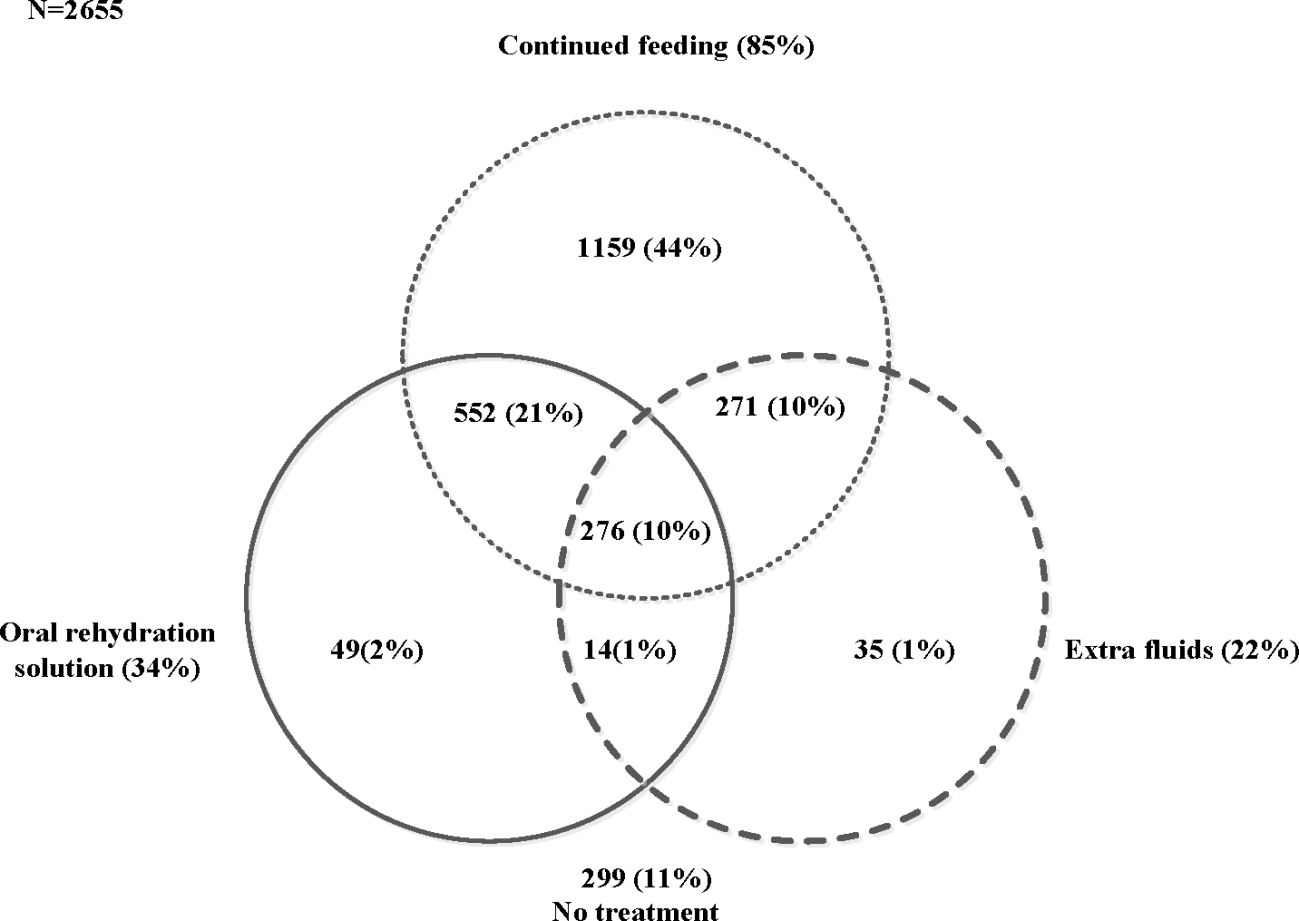
Number and percentage of children who received ORS, continued feeding and/or extra fluids during diarrhoea in Nepal (2001–2011).

The Venn diagram shows all the three treatment approaches in Nepal, 2001–2011 (Fig 2). In the figure, 21%, 10% and 1% of children aged 0–59 months were given ORS and continued feeding, continued feeding and extra fluids, and ORS and extra fluids, respectively. 10% of children were given ORS, extra fluids and continued feeding and 11% of children did not use any of three treatment approaches.

### Trends in diarrhoea management approaches

**Fig 3:**
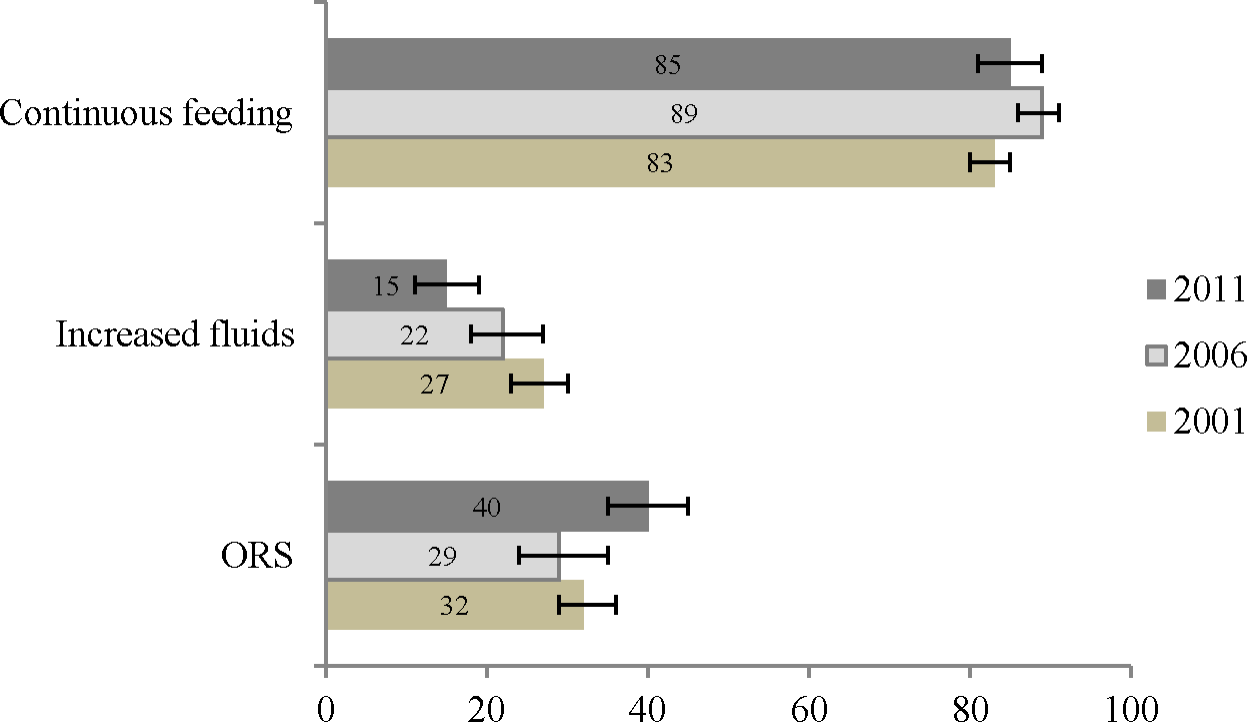
Trends in prevalence of Oral Rehydration Solution (ORS), increased fluids, and continued feeding during childhood diarrhoea in Nepal (2001–2011).

We found that the prevalence of ORS use increased significantly from 29% in 2006 to 40% in 2011, whereas the use of extra fluids decreased significantly from 27% in 2001 to 15% in 2011 (Fig 3). Over the 10 years, the prevalence of continued feeding fluctuated from between 83% in 2001, 89% in 2006, and 85% in 2011, and the prevalence of continued feeding significantly increased by 6% in 2006 compared to 2001, and a non-statistically significant reduction of 4% in 2011 compared to 2006.

### Univariate and multivariate logistic analyses

**Fig 4:**
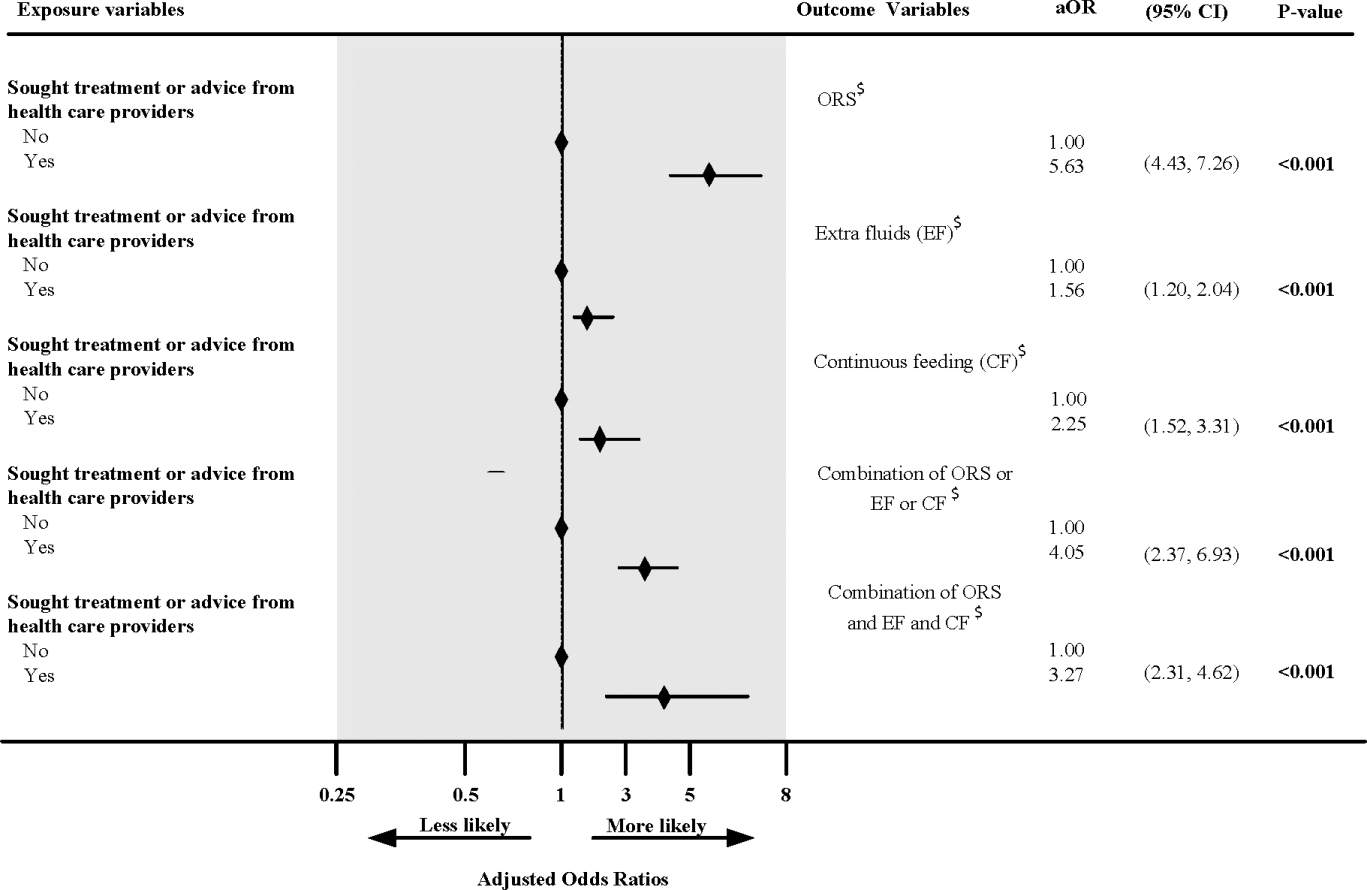
Impact of health service use on diarrhoea treatment approaches among children aged 0–59 months in Nepal (2001–2011). ^$^Adjusted for type of residence, ecological zone, geographical region, mother education, mother literacy level, father education, mother age, religion, parity, sex of child, mother working status, mother occupation, household wealth, months of data collection, age of child, and use of health service during diarrhoea.

Univariate analyses revealed that caregivers who sought treatment or advice from the health care providers were significantly more likely to use at least one the three prescribed approaches, ORS or extra fluids or continued feeding (OR 3.64, 95% CI 2.24, 5.90) for the treatment of childhood diarrhoea compared to those who did not seek treatment or advice from the health care providers (S1 Table). We also found increasing use of all approaches, ORS (OR 5.48, 95% CI 4.36, 6.88), extra fluids (OR 1.67, 95% CI 1.30, 2.15), continued feeding (OR 2.04, 95%CI 1.49, 2.80) among caregivers who sought treatment or advice from health care providers compared to those who did not seek treatment or advice from health care providers. This result was also found for the combination of all treatment approaches, ORS and extra fluids and continued feeding (OR 3.44, 95% CI 2.52, 4.72). These results remained significant in the adjusted model: ORS or extra fluids or continued feeding (aOR 4.05, 95% CI 2.37, 6.93), ORS (aOR 5.63, 95% CI 4.43, 7.26), extra fluids (aOR 1.56, 95% CI 1.20, 2.04), continued feeding (aOR 2.25, 95% CI 1.52, 3.31), ORS and extra fluids and continued feeding (aOR 3.27, 95% CI 2.31, 4.62) (Fig 4).

There were several socio-demographic variables that were important determinants of use of the management approaches, including geographical area of residence, maternal education, paternal education, and household wealth index. The use of all treatment approaches was significantly higher among mothers residing in the eastern geographical region (aOR 1.88, 95% CI 1.09, 3.26) compared to mothers residing in the central geographical region, mothers with primary education (aOR 1.48, 95% CI 1.00, 2.19) or secondary to higher education (aOR 2.93, 95% CI 1.86, 4.63) compared to uneducated mothers, fathers with primary education (aOR 1.55, 95% CI 1.11, 2.17) or secondary to higher education (aOR 1.58, 95% CI 1.01, 2.49) compared to uneducated fathers, and the family with middle household wealth index (aOR 2.25, 95% CI 1.53, 3.32) or rich household wealth index (aOR 1.63, 95% CI 1.02, 2.60) compared to the family with poor household wealth index. Among mothers who reported childhood diarrhoea, the majority (24%) of the illiterate women were from Central Geographical Region compared to 14% in Eastern, 7% in Mid-western, 6% in Far-western, and 8% in Western Geographical Region.

## Discussion

Despite the established benefits of using ORS, extra fluids and continued feeding as strategies to reduce mortality and morbidity from diarrhoea [6, 7, 11], optimal management is still not universally adopted in Nepal. Our study found that caregivers who sought treatment or advice from the health care providers were more likely to use treatment approaches (ORS, extra fluids and/or continued feeding) for childhood diarrhoea compared to those who did not seek treatment or advice from the health care providers.

The knowledge of ORS is almost universal in Nepal [27, 20]. However, this study found that the aggregate prevalence of ORS was about one-third whereas approximately a quarter of the sample reported extra fluids. The difference between knowledge and practice may be as a result of socio-cultural values related to help seeking for childhood illness and different ethnical views about causes and consequences of diarrhoea which prevented mothers from accessing modern healthcare for management of diarrhoea[28]. Similarly, the study conducted in a rural part of Nigeria reported that large improvements in knowledge of Oral Rehydration Therapy (ORT) from about 6 to 47 percent did not translate into practice with only 10 percent using ORT during diarrhoeal episodes[29]. The gap between knowledge and ORS use was also reported in hospital based studies in India, and a hospital based cross-sectional study in Pakistan [30–32]. These studies recommended that community outreach programs[30], widespread health education for mothers[31], and awareness programs around diarrhoea management approaches can bridge the knowledge and practice gap among mothers.

Our study found that attending a health service improved the management of diarrhoea (Fig 4). We found a strong association between health service utilization and the use of ORS, extra fluids and/or continued feeding during diarrhoea treatment. This finding is similar to a study conducted in a poor neighbourhood in Nicaragua[33] which reported that that ORS use was significantly associated to health service utilization. The authors concluded that mothers did not use ORS until they visited the health professional. A study conducted in Uganda by Nanyonjo et al [34] revealed that Integrated Community Case Management attendance for diarrhoea was associated with ORS use. It is useful to note that studies from developing countries including Nepal have documented some beliefs such as high fluid intake worsened diarrhoea, children with diarrhoea should be given only water due to teething, some forms of diarrhoea require traditional methods like exorcism, and intensity of diarrhoea is decreased with food restriction [28, 35–37]. These beliefs might have negatively affected the management of diarrhoea. Hence, knowledge, attitude and practice, which are deeply rooted into local cultural values and norms, appear to be important and health professionals can change cultural beliefs and improve knowledge about the use of diarrhoea treatment approach. For example, health care workers proving information to caregivers were found to improve the use of ORS and extra fluids as indicated in a study conducted in Ethiopia[38]. The government of Nepal policy to provide counselling on continued feeding during diarrhoea while patients are sought treatment or advice from health care providers may have contributed for the significant role for patient’s adherence to continued feeding[15]. Hence, widespread heath education for caregivers as well as awareness programs to improve knowledge, attitude and practice could bridge the widening gap and motivate caregivers to use recommended treatment approaches during diarrhoea. Past studies [34, 38, 39] have also suggested that a proper interaction between health worker and patient/care giver is crucial to improve the rate of treatment use and recovery. These findings suggest the need for educating family members particularly, mothers, their husbands and mother in-law about the importance and how to adequately use complementary and ORS to the children during diarrhoea.

Our study found that caregivers of children aged 0–59 months who received treatment or advice from health care provider reported higher odds of practicing ORS compared to other diarrhoea management approaches (extra fluids, continued feeding, combination of ORS & extra fluids & continued feeding, and combination of ORS or extra fluids or continued feeding). This finding was supported by a recent systematic review that estimated the effectiveness of ORS on diarrhoea mortality and the study concluded that ORS is more effective in reducing mortality related childhood diarrhoea in home, community and facility settings[11].

The major strengths of this study include the use of a nationally representative pooled sample, with an average response of 97%, use of standardised survey questionnaires, and the adjustment for the cluster sampling design with sampling weight. However, findings from this study do not accurately capture changes in diarrhoea management in Nepal as the Nepalese government introduced zinc in the treatment protocol for the management of childhood diarrhoea in 2007. We could not retain the use of the zinc variable into our pooled study because NDHS 2001 had no zinc related data. Similarly, the use of antibiotics or other medicine, an important confounder was excluded from this study due to no observations recorded in 2001 NDHS dataset.

## Conclusions

Our study concludes that caregivers of children aged 0–59 months of age are more likely to adhere with all three treatment approaches if they seek care or advice from health care providers. However, community based complete intervention packages such as the use of ORS, extra fluids and continued feeding are needed to further manage childhood diarrhoea in Nepal and such intervention should target caregivers of children from low socioeconomic disadvantaged group.

## Supporting information

S1 Table Unadjusted odd ratios (95% confidence interval (CI)) for the use of ORS, extra fluids and/or continued feeding during childhood diarrhoea in Nepal, NDHS 2001–2011. (DOCX)
