# Supplementary material for: Association between health service use and diarrhoea management approach among caregivers of under-five children in Nepal
Source: PLoS One. 2018 Mar 1;13(3):e0191988. doi: 10.1371/journal.pone.0191988 (PMC5832205; doi:10.1371/journal.pone.0191988)
Supplement: S1 Table — (DOCX) [file pone.0191988.s001.docx]

|  | ORS | Extra fluids (EF) | Continued feeding (CF) | Combination of ORS & EF & CF | Combination of ORS or EF or CF |
| --- | --- | --- | --- | --- | --- |
| Study variables | OR(95% CI) | OR(95% CI) | OR(95% CI) | OR(95% CI) | OR(95% CI) |
| **Type of Residence** |  |  |  |  |  |
| Rural | Reference | Reference | Reference | Reference | Reference |
| Urban | 1.32(1.02, 1.70)* | 1.40(0.99, 1.99) | 1.15(0.80, 1.65) | 1.72(1.13, 2.62)* | 1.16(0.74, 1.83) |
| **Ecological zone** |  |  |  |  |  |
| Mountain | Reference | Reference | Reference | Reference | Reference |
| Hill | 1.19(0.89, 1.60) | 1.37(1.02, 1.83)* | 1.01(0.68, 1.51) | 1.92(1.30, 2.82)* | 0.80(0.47, 1.36) |
| Terai | 1.39(1.04, 1.85)* | 0.57(0.41, 0.79)* | 0.49(0.35, 0.69)** | 0.94(0.62, 1.41) | 0.39(0.25, 0.62)** |
| **Geographical region** |  |  |  |  |  |
| Central | Reference | Reference | Reference | Reference | Reference |
| Eastern | 1.53(1.09, 2.14)* | 1.96(1.33, 2.87)* | 1.40(1.01, 1.94)* | 2.19(1.31, 3.68)* | 1.46(0.99, 2.16) |
| Western | 1.09(0.77, 1.54) | 1.72(1.21, 2.45)* | 1.33(0.90, 1.96) | 1.55(0.93, 2.59) | 1.49(0.95, 2.33) |
| Mid-western | 1.36(0.93, 2.00) | 1.82(1.26, 2.65)* | 1.44(0.92, 2.24) | 1.54(0.83, 2.88) | 1.91(1.07, 3.40)* |
| Far-western | 1.38(1.00, 1.91)* | 1.37(0.99, 1.91) | 1.88(1.24, 2.84)* | 1.51(0.95, 2.41) | 2.20(1.38, 3.51)* |
| **Mother education** |  |  |  |  |  |
| No education | Reference | Reference | Reference | Reference | Reference |
| Primary | 1.17(0.88, 1.55) | 1.69(1.28, 2.22)** | 0.99(0.69, 1.40) | 1.87(1.28, 2.73)* | 1.09(0.73, 1.63) |
| Some secondary to higher | 1.93(1.46, 2.55)** | 3.10(2.39, 4.02)** | 1.37(0.96, 1.96) | 3.91(2.76, 5.54)** | 1.81(1.56, 2.84)* |
| **Mother literacy level** |  |  |  |  |  |
| Cannot read at all | Reference | Reference | Reference | Reference | Reference |
| Able to read | 1.62(1.30, 2.03)** | 2.50(1.99, 3.13)** | 1.36(1.05, 1.75)* | 2.95(2.20, 3.95)** | 1.70(1.27, 2.28)** |
| **Father education** |  |  |  |  |  |
| No education | Reference | Reference | Reference | Reference | Reference |
| Primary | 1.43(1.14, 1.80)* | 1.92(1.51, 2.43)** | 1.44(1.06, 1.95)* | 2.41(1.74, 3.34)** | 1.49(1.06, 2.11)* |
| Some secondary to higher | 1.59(1.22, 2.06)* | 2.01(1.50, 2.68)** | 1.16(0.82, 1.64) | 2.91(1.98, 4.27)** | 1.06(0.72, 1.57) |
| **Mother age** |  |  |  |  |  |
| 30-49 | Reference | Reference | Reference | Reference | Reference |
| 20-29 | 1.51(1.18, 1.93)* | 1.14(0.89, 1.46) | 0.91(0.69, 1.22) | 1.25(0.90, 1.72) | 1.04(0.75, 1.45) |
| <20 | 1.42(0.94, 2.12) | 1.34(0.87, 2.08) | 0.36(0.24, 0.56)** | 1.21(0.72, 2.01) | 0.45(0.28, 0.72)* |
| **Religion** |  |  |  |  |  |
| Buddhist | Reference | Reference | Reference | Reference | Reference |
| Hindu | 1.22(0.84, 1.78) | 0.78(0.52, 1.18) | 0.88(0.49, 1.56) | 0.80(0.44, 1.45) | 0.98(0.50, 1.95) |
| Others | 1.14(0.69, 1.90) | 0.59(0.35, 1.00) | 0.82(0.41, 1.64) | 0.62(0.27, 1.39) | 0.75(0.34, 1.67) |
| **Parity** |  |  |  |  |  |
| 6+ | Reference | Reference | Reference | Reference | Reference |
| (4-5) | 1.29(0.91, 1.82) | 1.06(0.69, 1.60) | 1.09(0.74, 1.60) | 1.03(0.59, 1.78) | 1.22(0.79, 1.89) |
| (2-3) | 1.69(1.20, 2.36)* | 1.21(0.84, 1.76) | 1.13(0.77, 1.65) | 1.50(0.88, 2.58) | 1.36(0.89, 2.07) |
| 1 | 1.95(1.37, 2.79)** | 1.81(1.20, 2.71)* | 1.30(0.87, 1.96) | 2.45(1.43, 4.20)** | 1.70(1.10, 2.60)* |
| **Sex of child** |  |  |  |  |  |
| Female | Reference | Reference | Reference | Reference | Reference |
| Male | 1.3(1.08, 1.56)* | 1.25(1.05, 1.49)* | 1.34(1.06, 1.70)* | 1.26(0.97, 1.65) | 1.49(1.13, 1.97)* |
| **Mother working status** |  |  |  |  |  |
| Currently working | Reference | Reference | Reference | Reference | Reference |
| Currently not working | 1.19(0.93, 1.51) | 0.75(0.56, 1.00) | 0.63(0.47, 0.85)* | 0.77(0.53, 1.12) | 0.65(0.46, 0.92)* |
| **Mother occupation** |  |  |  |  |  |
| Agriculture | Reference | Reference | Reference | Reference | Reference |
| Non- agriculture | 1.33(0.91, 1.96) | 1.57(1.04, 2.36)* | 1.45(0.78, 2.67) | 1.81(1.08, 3.02)* | 1.38(0.65, 2.92) |
| Not working | 1.21(0.94, 1.55) | 0.88(0.66, 1.19) | 0.68(0.50, 0.93)* | 0.99(0.66, 1.50) | 0.68(0.48, 0.96)* |
| **Household wealth index** |  |  |  |  |  |
| Poor | Reference | Reference | Reference | Reference | Reference |
| Middle | 0.90(0.71, 1.15) | 2.02(1.53, 2.67)** | 0.69(0.51, 0.94)* | 1.52(1.06, 2.20)* | 0.63(0.44, 0.89)* |
| Rich | 1.49(1.16, 1.91)* | 2.58(1.91, 3.48)** | 1.01(0.72, 1.43) | 2.37(1.63, 3.43)** | 1.00(0.66, 1.54) |
| **Months of data collection** |  |  |  |  |  |
| January- March | Reference | Reference | Reference | Reference | Reference |
| April- August | 0.77(0.60, 0.98)* | 0.87(0.66, 1.14) | 1.69(1.32, 2.15)** | 0.81(0.55, 1.20) | 1.61(1.19, 2.16)* |
| **Child age in months** |  |  |  |  |  |
| (0-11) | Reference | Reference | Reference | Reference | Reference |
| (12-23) | 2.40(1.83, 3.17)** | 1.86(1.41, 2.47)** | 15.72(10.27, 24.06)** | 2.99(1.83, 4.89)** | 19.66(11.16, 34.65)** |
| (24-59) | 2.13(1.62, 2.81)** | 1.75(1.33, 2.30)** | 45.9(26.77, 78.72)** | 2.68(1.76, 4.07)** | 61.11(29.32, 127.39)** |
| **Use of health service during diarrhoea** |  |  |  |  |  |
| No | Reference | Reference | Reference | Reference | Reference |
| Yes | 5.48(4.36, 6.88)** | 1.67(1.30, 2.15)** | 2.04(1.49, 2.80)** | 3.44(2.52, 4.72)** | 3.64(2.24, 5.90)** |

S1 Table. Unadjusted odd ratios (95% confidence interval (CI)) for the use of ORS, extra fluids and/or continued feeding during childhood diarrhoea in Nepal, NDHS 2001-2011

**: p<0.001; *: p<0.05
